# The importance of change communication and state motivation when adapting to changes

**DOI:** 10.1111/sjop.13066

**Published:** 2024-08-19

**Authors:** Lukasz Stasielowicz

**Affiliations:** ^1^ Osnabrück University Osnabrück Germany

**Keywords:** Adaptive performance, adaption to change, transition adaption, change communication, motivation

## Abstract

Employees often work in dynamic environments requiring adaptive performance (e.g., emergencies, clients from other cultures). To optimize change management, employee training, and personnel selection in organizations, researchers have focused on trait‐like predictors of adaption to change, such as personality traits or cognitive ability. The study (*N* = 300) shifts the focus to more proximal performance predictors – change communication and task‐related state motivation. Adaptive performance was modeled using latent growth models. Providing two change‐related hints, one at the beginning of the task and another directly after the change, mitigated performance impairment observed directly after the change. Moreover, this advantage largely persisted throughout the later stages of the task. In contrast, a single hint at the beginning of the task did not substantially facilitate adaption. Finally, task‐related state motivation was linked to better performance on the subsequent measurement occasion. Organizations might minimize change‐induced losses by deploying adequate change communication and maintaining employee motivation.

## INTRODUCTION

Since job characteristics have changed over the years (Wegman, Hoffman, Carter, Twenge & Guenole, 2018), and a typical modern workplace is a dynamic environment (Ryan & Ployhart, 2014), employees must adapt to changes, such as new coworkers or learning new technologies. Failure to show adaptive performance can lead to organizational costs. For example, clients unsatisfied with longer waiting times and higher error rates might translate into financial losses for the organization. Personal consequences for employees are also possible, including reduced promotion prospects and lack of performance bonuses. Thus, it is not surprising that many organizations launched their change management departments or followed instructions from consulting firms to deal with change processes appropriately (Jick & Sturtevant, 2017). Organizations might also implement employee training to promote the general capacity to show adaptive behavior across different situations – adaptability or adaptive expertise (Bohle Carbonell, Stalmeijer, Könings, Segers & van Merriënboer, 2014; Kua, Lim, Teo & Edwards, 2021; Wallin, Nokelainen & Mikkonen, 2019; Ward, Gore, Hutton, Conway & Hoffman, 2018). Finally, adaptive performance can be considered in personnel selection (Ryan & Ployhart, 2014).

To facilitate dealing with change, organizations and researchers try to identify predictors of adaptive performance. Hitherto, multiple variables were examined as predictors, and some of these relationships were investigated meta‐analytically. Examples include personality traits (Huang, Ryan, Zabel & Palmer, 2014; Woo, Chernyshenko, Stark & Conz, 2014), goal orientation (Stasielowicz, 2019), and cognitive abilities (Stasielowicz, 2020). Although such trait‐like variables might be indirectly related to performance in a specific situation, it is also important to consider predictors located spatiotemporally closer to adaptive performance (Jundt & Shoss, 2023; Jundt, Shoss & Huang, 2015). After all, current emotional, cognitive, and motivational processes can influence performance through feelings of stress, devoting attention to certain aspects of the task, or changes in confidence based on recent experiences. However, researchers usually examine distal predictors of individual adaptive performance rather than proximal predictors. This study aims to help close this research gap by investigating the relevance of change communication and state motivation to the adaption process in a dynamic environment. Since many employees experience changes at work, the findings will inform organizational policies in various areas, such as change management and employee training. To what extent does change‐related information help people mitigate change‐induced losses? How long‐lasting are the benefits of change communication? How much does task‐related state motivation matter for performance in dynamic contexts? The following paragraphs provide a brief overview of the adaptive performance concept, followed by a description of the research questions that will be examined in the current study.

### Adaptive performance

According to Pulakos, Arad, Donovan & Plamondon (2000, p. 615), adaptive performance refers to “altering behavior to meet the demands of a new situation, event, or set of circumstances.” Similar to other performance dimensions (Campbell & Wiernik, 2015), adaptive performance is usually expected to contribute to the organization's goals. To account for the fact that there are different types of change, multiple adaptive performance dimensions were proposed (Pulakos *et al*., 2000): (1) solving problems creatively; (2) dealing with uncertain or unpredictable work situations; (3) learning new tasks, technologies, and procedures; (4) demonstrating interpersonal adaptability; (5) demonstrating cultural adaptability; (6) demonstrating physically oriented adaptability; (7) handling work stress; and (8) handling emergencies or crisis situations. The proposed dimensions demonstrate the relevance of this construct in many work areas. Adaptive performance can be required in the office (e.g., learning new software or dealing with new colleagues) and in more extreme environments or situations, such as healthcare emergencies (Fernandez, Rosenman, Plaza‐Verduin & Grand, 2022), air‐traffic control during a terrorist attack (Foster, Plant & Stanton, 2019) or astronauts dealing with changing conditions (Bartone, Krueger & Bartone, 2018). Notably, different jobs may require distinct forms of adaptive performance. For example, physical adaptability is more relevant in the military than in accounting.

All the mentioned adaptive performance dimensions can be quickly assessed by using questionnaires (Charbonnier‐Voirin & Roussel, 2012; Griffin & Hesketh, 2003). However, more objective measures are also available, and this approach will be adopted in the current study. Objective adaptive performance scores are usually collected by asking people to complete a task, which might tap into several dimensions, such as dealing with uncertain situations and learning new procedures. The task requirements change suddenly, and participants need to modify their behavior. One advantage of this task‐change paradigm is that it enables a nuanced view of the adaption process (Jundt & Shoss, 2023). Immediately after the change, a performance impairment is usually observed, followed by gradual improvements in the later stages.

According to the model proposed by Jundt and Shoss (2023), the adaption process depends on detecting or anticipating change, identifying change‐related demands, choosing and implementing strategies to deal with change, and possibly revising strategies through learning. Since adaption can be viewed as a process, some researchers use longitudinal modeling to distinguish between different adaption indicators (Bliese & Lang, 2016; Lang & Bliese, 2009). This approach will also be used in this study to examine objective task scores.

The current study's main goal is to examine the relevance of some proximal predictors to the adaption process. Multiple emotional, cognitive, and motivational variables can promote or impair the adaption process at different stages (Jundt & Shoss, 2023). Assessing such variables shortly before and during the task helps clarify their relevance. However, such proximal predictors of performance were rarely investigated in previous studies based on the task‐change paradigm. Researchers often focused on trait‐like distal predictors of adaptive performance, such as personality factors (Huang *et al*., 2014; Woo *et al*., 2014), goal orientation (Stasielowicz, 2019), and cognitive ability (Stasielowicz, 2020). Although distal variables might indirectly impact adaptive performance in a specific situation, it is also important to examine variables with more direct effects on the adaption process (Jundt & Shoss, 2023; Jundt *et al*., 2015).

Although distal predictors of adaptive performance were examined more extensively in the past, some research findings involve proximal predictors. To illustrate, metacognitive predictors such as planning, goal monitoring, and evaluation have been linked to better adaptive performance (Christian, Christian, Pearsall & Long, 2017; Jundt *et al*., 2015). This pattern is not surprising considering the conceptual similarities between metacognitive activities and the different stages of the adaption process; metacognitive activities such as planning and goal monitoring can promote adaptive performance through choosing and implementing good strategies. Furthermore, preparing for and engaging with the task is conducive to learning and knowledge acquisition, which can also benefit adaptive performance (Jundt & Shoss, 2023; Jundt *et al*., 2015). Researchers have also examined other proximal predictors, including self‐efficacy and interest (Jundt *et al*., 2015). However, as Jundt and colleagues note, many findings involving proximal predictors could be obfuscated because the predictors were assessed only before but not during the task. Measuring proximal predictors and performance on several occasions is important in dynamic environments because stress, attention, motivation, and other aspects might change during the task. The relationship between predictor and performance might also change.

More recently, researchers began to include time‐varying covariates when investigating performance trajectories in the task‐change paradigm (Jorgensen, Day, Huck, Westlin, Richels & Nguyen, 2021). In one study, higher affect variability was linked to worse adaptive performance (Richels, Day, Jorgensen & Huck, 2020), which confirms that emotional processes are relevant to the adaption process (Jundt & Shoss, 2023). The study's design follows this approach by modeling the relevance of proximal predictors for the performance trajectory rather than the aggregate performance score. Specifically, the current study includes proximal predictors of adaptive performance, which have not been examined using the nuanced process approach and objective performance data – change communication and state motivation. Crucially, motivation will be assessed several times during the task to investigate its relevance at different stages of the adaption process. In addition, some participants will be informed that the task requirements will change or have already been changed, which will enable estimating to what extent change communication facilitates adaption.

### Change communication and adaptive performance

Since modern workplaces are dynamic, organizations must know to what extent changes should be communicated. Change communication refers to informing employees about imminent or current change. Communicating change could reduce errors and increase adaption speed, resulting in financial benefits for organizations. Previous findings indicate that change communication and information are important in the context of organizational changes (Oreg, Vakola & Armenakis, 2011). In one longitudinal study, informing people about organizational changes and their implications, such as having different colleagues at work or having to take part in training, was positively related to self‐reported performance after the change (Van den Heuvel, Demerouti, Bakker & Schaufeli, 2013). A similar pattern was observed in other studies using subjective performance ratings (Parent, Sullivan, Hardway & Anthony Butterfield, 2012; Petrou, Demerouti & Schaufeli, 2018).

While general change communication was studied extensively, there is surprisingly little research based on more objective performance data and examining communication of changed requirements in a single task rather than several areas simultaneously. Therefore, the first goal of the study is to determine whether informing people about changes in a particular task can improve adaptive performance on this task. The already mentioned task‐change paradigm is particularly well‐suited to examine this research question. When using the task‐change paradigm, people are asked to complete a specific task. A repetitive character of the task enables participants to familiarize themselves with it. The initial learning phase is effortful and requires much attention. However, people can automate their behavior through repeated exposure to similar stimuli. Specifically, people can internalize stimulus–response associations (Ackerman, 1988). Specific behaviors can then be associated with a mental representation of a particular problem, which means that routines can be established (Betsch, Haberstroh & Höhle, 2002).

However, routines might be maladaptive if the environment changes (Bröder & Schiffer, 2006). In dynamic situations, it might be helpful to not over‐rely on past decisions (McCormick, Cheyette & Gonzalez, 2022; Rakow & Miler, 2009). Importantly, the task‐change paradigm enables one to study initial experiences' stickiness in dynamic environments. After a number of trials or after a certain period, the task requirements change, and old routines might need to be modified to maintain good performance levels. Providing a hint about the change could help people realize faster that they cannot rely on old routines and need to adjust their behavior. Relatedly, there is evidence that valuing recent experiences more than older experiences can be helpful in dynamic situations (Konstantinidis, Harman & Gonzalez, 2022). Providing a hint could help people focus on recent experiences and quickly identify changes. This is important because change detection or anticipation and preparing plans are the first stages of the adaption process (Jundt & Shoss, 2023).

Hitherto, the relevance of change communication to a specific task was rarely examined. In one study (Bröder & Schiffer, 2006), a hint about change was given directly after the change. The task involved choosing stocks based on cues, and the optimal strategy changed after 80 trials. Some participants were informed that the stock market would change (Germany vs. the USA) and that the environments could differ. However, the hint did not substantially affect the strategies in the post‐change phase. Therefore, the study will use a more informative hint to facilitate adaption. Specifically, some participants will receive information that the importance of task cues changed due to an external event. This design will help investigate the first research question.

**Research question 1.** To what extent does change communication facilitate adaptive performance?

**Fig. 1:**
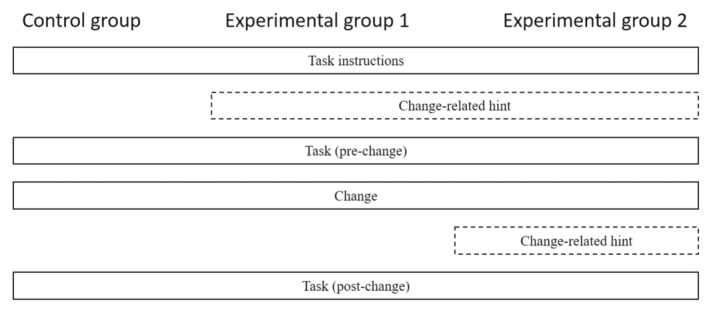
Illustration of the experimental design in the study.

To examine this research question, three conditions will be implemented in this study. The experimental design is illustrated in Fig. 1: (1) one group will receive no change‐related information (control condition); (2) some people will be provided with a hint at the beginning that task requirements might change due to external conditions (1st experimental condition); and (3) one group will receive both the initial hint and a hint directly after the change that the importance of the task cues changed (2nd experimental condition). Based on the skill acquisition literature (Ackerman, 1988; Betsch *et al*., 2002) and previous adaptive performance studies (Lang & Bliese, 2009; Niessen & Lang, 2021), it is expected that the repetitive nature of the adaptive performance task will lead to automation and establishing of routines. Therefore, changing task requirements should result in performance impairment. However, change communication could speed up the adaption process by helping people recognize more quickly that old routines are ineffective in the changed environment. Change detection and identification of change‐related demands are regarded as critical first steps of the adaption process (Jundt & Shoss, 2023).

Receiving change‐related information could influence emotional, cognitive, and motivational processes. To illustrate, realizing more quickly that a change occurred could prompt people to put effort into identifying better strategies sooner. Change communication could also stimulate metacognition (e.g., planning and goal monitoring), which has been linked to better adaptive performance in previous studies (Jundt *et al*., 2015). Providing change‐related information will not necessarily directly improve activities regarded as late stages of the adaption process, such as learning and revising strategies (Jundt & Shoss, 2023). Still, it could enable reaching those stages faster than without change‐related information.

Crucially, using the task‐change paradigm enables a nuanced investigation of the adaption process and its predictors. Specifically, one can examine the extent of performance impairment directly after the change (i.e., transition adaption) and the (re‐)learning rate in the post‐change phase (i.e., reacquisition adaption). Change communication could theoretically improve both transition adaption and reacquisition adaption. A direct hint after the change could prompt people to adjust their strategies faster, which could mitigate short‐term performance impairment and improve the subsequent learning rate.

The present study extends the previous research by manipulating the timing and repetition of change communication. Bröder & Schiffer (2006) used a moderately informative hint only directly after the change. In contrast, the first experimental condition in the study will receive one hint well in advance (i.e., at the beginning of the task), and the second experimental condition will receive an additional informative hint directly after the change (i.e., information that the importance of two specific task cues has changed). A single hint at the beginning of the task could help increase change readiness and post‐change task effort. Providing an additional informative hint directly after the change in the 2nd experimental condition is expected to help break the routines faster, resulting in better transition adaption.

The positive effect of change communication might extend into the later stages of adaption. Specifically, positive effects of change communication could manifest not only as reduced losses immediately after the change (i.e., transition adaption) but also faster learning after the initial change‐related performance impairments (i.e., reacquisition adaption). Although studies using self‐reported performance data usually do not distinguish between different adaption stages, one could argue that previous studies' broad conceptualization of the adaption process encompasses both immediate and medium‐term adaption. In those studies, change communication is often perceived as being generally helpful in adapting to changes (Parent *et al*., 2012; Petrou *et al*., 2018; Van den Heuvel *et al*., 2013). Therefore, it seems plausible that the positive effects of change communication extend into the later stages of the adaption process. However, a more nuanced empirical approach is needed to confirm this. Therefore, the study will use objective performance data to examine the effects of change communication on transition and reacquisition adaption.

### Task‐related state motivation and performance

In addition to examining the relevance of change communication to the adaption process, the study also investigates to what extent task‐related state motivation is related to subsequent performance. According to the adaption process model (Jundt & Shoss, 2023), not only cognitive and emotional but also motivational processes can be relevant during adaption. To illustrate, highly motivated people could expect a high probability of success on the upcoming task trials and perceive the task as fun, which could impact the actual performance.

**Research question 2.** To what extent does task‐related state motivation affect performance on an adaptive performance task?

Examining motivational aspects in dynamic environments can be fruitful, as performance often depends on motivation (Van Iddekinge, Aguinis, Mackey & DeOrtentiis, 2018). Motivated people might be more willing to put more effort into performing well by directing more energy to the task, increasing the intensity of their actions and persistence (Van Iddekinge, Arnold, Aguinis, Lang & Lievens, 2023). According to the process model of adaptive performance, motivational aspects could be relevant at different stages of the adaption process (Jundt & Shoss, 2023); high motivation could be beneficial to identifying change‐related demands, developing strategies, learning, and revising strategies.

Thus, it is not surprising that the relationship between motivational constructs and adaptive performance was examined in the past. One research team assessed task motivation after participants completed an air traffic control simulation. Across two studies, task motivation was only related to some performance indicators (Niessen & Lang, 2021). Mixed findings were also reported in a meta‐analysis assessing the relationship between adaptive performance and the motivational constructs of learning goal orientation and performance goal orientation (Stasielowicz, 2019). While both the motivation to learn and the motivation to show good performance were related to self‐reported adaptive performance, there was no substantial relationship with objective performance data. However, it is important to note that motivational aspects were usually assessed only once, for example, before the task commenced or after completing it. Furthermore, researchers often used trait measures of motivation. Since state motivation seems more relevant than trait motivation to certain kinds of performance (Van Iddekinge *et al*., 2018), examining state motivation in a dynamic context could be fruitful.

Therefore, the study attempts to close the research gap by assessing task‐related motivational states on three occasions during the task. Specifically, motivation will be assessed before the task, shortly after the task begins, and shortly after the change occurs. This approach keeps the number of distractions during the task at a minimum while enabling a more nuanced perspective than a single motivation assessment. Based on the findings pertaining to other performance facets (Van Iddekinge *et al*., 2018), the theoretical relevance of motivation to the different stages of the adaption process (Jundt & Shoss, 2023), and because of the temporal proximity of assessing state motivation and performance, task‐related motivation is expected to be related to performance on the adaptive performance task in the study. Specifically, higher motivation is expected to improve performance on the next task block.

## METHODS

### Procedure

The data were collected online. The study link was sent to a professional platform enabling paid large‐scale data collection, and it was also distributed through free channels to reach as many occupationally active people as possible. After providing informed consent, participants answered demographic questions. Solely people over 18 years old received additional questions: job complexity, adaptive performance questionnaire, current affect, and two measures of task motivation (a short scale and a single‐item measure). Next, participants were asked to complete a task that measured their adaptive performance objectively.

At the beginning of the task, participants were asked to imagine receiving training as new bank employees. The goal of the training was to prepare them to make future stock acquisition recommendations to their clients. Participants had to assess the potential (0 = low, 30 = high) of various stocks by using two indicators: creditworthiness rating provided by a professional agency (1 = low creditworthiness, 4 = very high creditworthiness) and company's profit in the previous year (1 = ca. 10% of the revenue, 4 = ca. 40% of the revenue). Participants saw all 16 possible combinations of the two indicators (e.g., creditworthiness = 2, profit = 3). Six sets were presented (96 stimuli), with three sets in the pre‐change phase and three in the post‐change phase.

**Fig. 2:**
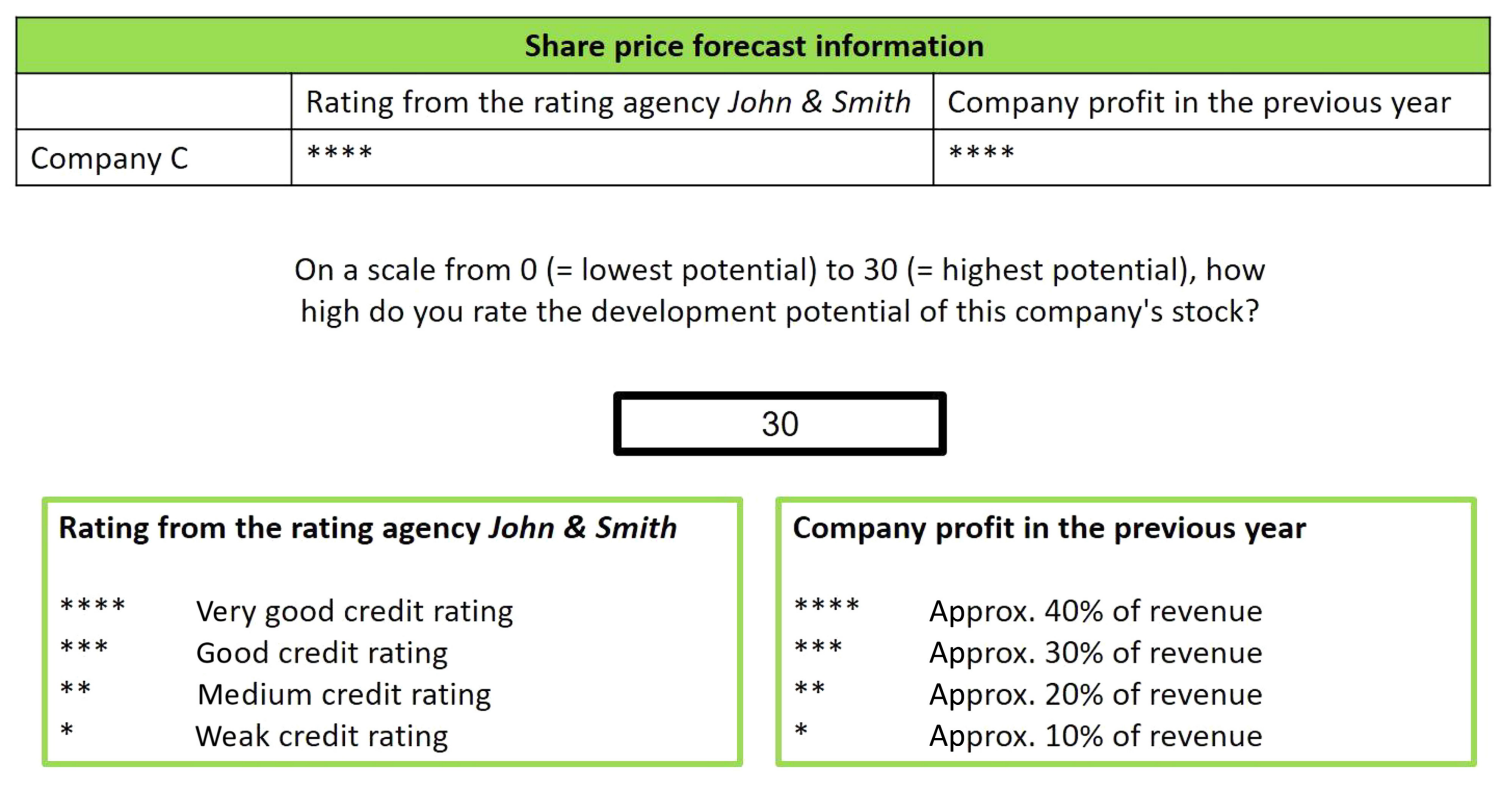
Example item of the task used to measure adaptive performance objectively. The item was shown in German. Participants rated (0–30) the company's share price development potential based on two indicators: creditworthiness rating from a professional agency and company profit. Initially, company profit was more important than the rating agency's opinion. However, the importance of the indicators changed during the task to enable measuring adaptive performance. In the post‐change phase, the creditworthiness rating was more important.

To familiarize the participants with the task, a text description and an example item were provided (see Fig. 2). The example item contained information about a fictitious company C, with maximal values of both indicators (i.e., creditworthiness rating and company profit). The participants were informed that the highest share price potential (i.e., 30) was the correct response and were asked to enter this value. After submitting the answer, participants were informed whether their rating was accurate.

Depending on the condition, some participants received change‐related information. Participants were randomly allocated to one of three conditions to examine to what extent change communication facilitates adaptive performance: (1) in the control condition, there was no change communication; (2) in the first experimental condition, potential change was communicated after showing the example item by pointing out that the importance of the indicators can change due to a stock market crash; and (3) in the second experimental condition change was communicated both after the example item and directly after the task requirements changed. Specifically, in the last condition, participants were informed after completing the third block of stimuli that a stock market change had occurred and that the importance of indicators (i.e., creditworthiness rating and company profit) would change.

All participants were given three sets of trials to learn the task. In the pre‐change phase, the profit indicator was more important than the creditworthiness indicator. The underlying equation was not disclosed, but participants could use the feedback about the response accuracy to optimize their performance. After completing the third task block, the indicator weights changed. Only participants in the second experimental condition were explicitly informed about the change. However, the specific weights of the indicators were not disclosed. Nonetheless, participants still received feedback after each response. They could use this information to improve their performance.

Between certain task blocks, various variables were assessed. Following the first task block, participants were asked about the strategies used during the task. In addition, the current affect and motivation were measured for the second time. Participants then returned to the main task and completed blocks 2 to 4. Next, the strategies were assessed for the second time, and the current affect and motivation were measured for the third and final time. Participants completed the main task (blocks 5 and 6) and answered additional questions about their strategies, aids (e.g., pen and paper), and whether they noticed the task change after the third task block.

Throughout the study, attention checks were used to minimize the risk of low data quality. Attention checks contained instructions asking the participants to make a particular choice, such as clicking the “next” button rather than answering filler questions. Participants in the panel sample received 7.8€ for completing the study, and other participants were given the option to choose between a draw (four 20€ vouchers), a course credit, or to reject compensation. Some of the mentioned variables were collected for undergraduate projects and are not considered in this article. Further information about additional measures can be found in the codebook at https://osf.io/x9dj5/. The main instruments are described in the next section.

### Measures

#### Motivational state

Three items were used to assess the motivational state using a seven‐point scale (1 = does not apply, 7 = applies). The participants were asked to rate the probability of success and positive valence when completing the adaptive performance task. An example item is “I'm sure I will find the correct solution” (Vollmeyer & Rheinberg, 2000). The motivational state was assessed before the task (*α* = 0.71, 95% CR [0.65–0.76]), before the second task block (*α* = 0.75, 95% CR [0.70–0.80]), and before the fifth task block (*α* = 0.75, 95% CR [0.70–0.80]).

#### Adaptive performance

The current implementation of the task‐change paradigm within the stock task (see Fig. 2) enabled measuring adaptive performance objectively. The participants had to rate the share price potential (0–30) based on the two indicators (i.e., creditworthiness rating and company profit). Task requirements changed after a certain number of trials to assess how participants adapt to change. In the pre‐change phase, the share price potential could be estimated using the following undisclosed equation:

$$\text{Share price potential}=\begin{array}{l} 4\times \text{Agency}\_\text{Rating}+6\\ \times \text{Company}\_\text{Profit}–10 \end{array}$$

Accordingly, a creditworthiness rating of 2 and a company profit of 4 corresponded to a share price potential of 22 (4 × 2 + 6 × 4–10).

After the change, the creditworthiness rating was more important than the profit indicator. The underlying equation was changed to:

$$\text{Share price potential}=\begin{array}{l} 7\times \text{Agency}\_\text{Rating}+3\\ \times \text{Company}\_\text{Profit}–10 \end{array}$$

To illustrate, a creditworthiness rating of 2 and a company profit of 4 corresponded to a share price potential of 16 (7 × 2 + 3 × 4–10).

The individual responses were used to compute the performance scores. The share price potential estimates provided by the participants were subtracted from the actual share price potential. Absolute differences were used because share price overestimation and underestimation were considered equally bad. The average absolute difference was computed for each set of the 16 stimuli. The average scores could theoretically vary between 0 (i.e., participant's response = real share price potential) and approximately 22 (i.e., the responses to all stimuli were as far as possible from the actual share price potential).

### Statistical analyses

Latent growth modeling was used to examine the role of change communication and state motivation in a dynamic environment. The respective analyses were carried out using the R package blavaan (0.4–7) for Bayesian latent variable analysis. The Bayesian approach enables one to incorporate prior statistical or field‐specific knowledge into the analysis (Kruschke & Liddell, 2017). For example, even before conducting the analyses, researchers know that negative variances of observed and latent variables are impossible and large effects or factor loadings are unlikely. This prior knowledge is combined with the participants' data to estimate the parameters of interest, such as regression coefficients. The distribution of the estimates is called posterior distribution, and it often relies on the Markov chain Monte Carlo (MCMC) method.

The underlying algorithm accepts or rejects candidate parameter values (e.g., regression coefficient = 0.23) using the priors and available data. Proposed values that are more congruent with priors and participants' data are more likely to be accepted. However, some chain values are not used in further analyses; the first ones are usually discarded because they are regarded as a warm‐up phase. Since the starting point of the chain is often chosen randomly, it might take a while before the algorithm starts exploring plausible values.

Usually, several independent chains are run to ensure that the results are robust. If different chains explore different regions, the results are unreliable, and the model needs to be modified. In contrast, if the chains mix well, then the post‐warm‐up values of all chains are used to describe the posterior distribution. The study uses four Markov chains for each latent growth model. Each chain contains 2,000 post‐warm‐up samples, yielding 8,000 posterior distribution samples in total. This distribution contains the most plausible parameter estimates based on prior knowledge and participants' data. The values of the posterior distribution are used to summarize the uncertainty of the estimates (Morey, Hoekstra, Rouder, Lee & Wagenmakers, 2016). Specifically, the 95% credibility interval limits are reported, containing the most plausible values.

Weakly informative priors are used in the study. Since effect sizes reported in the organizational literature are rarely large (Bosco, Aguinis, Singh, Field & Pierce, 2015), more prior probability is allocated to smaller than larger regression coefficients in the current study. Moreover, informative priors were chosen for correlations between variables describing the performance trajectory. To illustrate, people making only small improvements in the pre‐change phase are expected to make relatively small improvements in the post‐change phase, too. Thus, a positive correlation between the two learning rates is expected.

The performance trajectories will be modeled using the framework popularized by Lang & Bliese (2009) that was subsequently adopted in multiple studies (Howe, 2019; Niessen & Jimmieson, 2015; Niessen & Lang, 2021). The framework uses several change variables to model transitions by fixing these change variables to particular values at specific measurement occasions (Bliese & Lang, 2016). In the study, the change variables are modeled as latent variables with performance scores on the six task blocks as indicators. Table 1 shows how the loadings are fixed for specific measurement occasions in the study. The meaning of the change variables is summarized below.

**Table 1:** Coding of the latent variables describing adaptive performance trajectories. See text for explanations

	Measurement occasion					
	Pre‐change phase			Post‐change phase		
Change variable	1	2	3	4	5	6
Intercept	1	1	1	1	1	1
SA (Skill acquisition)	0	1	2	2	2	2
SA^2^ (quadratic SA)	0	1	4	4	4	4
TA (transition adaption)	0	0	0	1	1	1
RA (reacquisition adaption)	0	0	0	0	1	2
RA^2^ (quadratic RA)	0	0	0	0	1	4

The coding scheme presented in Table 1 is used to account for the dynamic nature of the task. Several variables are used to model the average performance trajectory. For each of those predictors, a regression coefficient will be estimated. These coefficients describe a specific part of the performance trajectory after accounting for the constraints imposed by the fixed loadings from Table 1. The intercept loadings are fixed to 1 across all measurement occasions. Combined with the other codings, this ensures that the regression coefficient for the latent intercept corresponds to the average performance at the first measurement occasion. The next change variable, skill acquisition (SA), reflects the learning rate in the pre‐change phase. To account for the fact that the learning rate can change during the task (e.g., substantial improvements at the beginning of the task and smaller improvements in the later stages), both a linear trend (SA) and a quadratic trend for skill acquisition (SA2) are included.

The remaining change variables are used to capture adaptive performance. Since the task requirements change after the third measurement occasion, a sudden performance drop is expected. The transition adaption variable (TA) reflects this immediate trajectory change. The chosen coding of SA (i.e., the digits stop increasing in the post‐change phase) ensures that the regression coefficient of the TA value can be interpreted as a performance drop between the third and fourth measurement occasions (Bliese & Lang, 2016). Finally, reacquisition adaption variables (RA and RA2) capture the linear and quadratic learning rate in the post‐change phase (i.e., the average improvement rates after the change‐induced performance impairment).

**Fig. 3:**
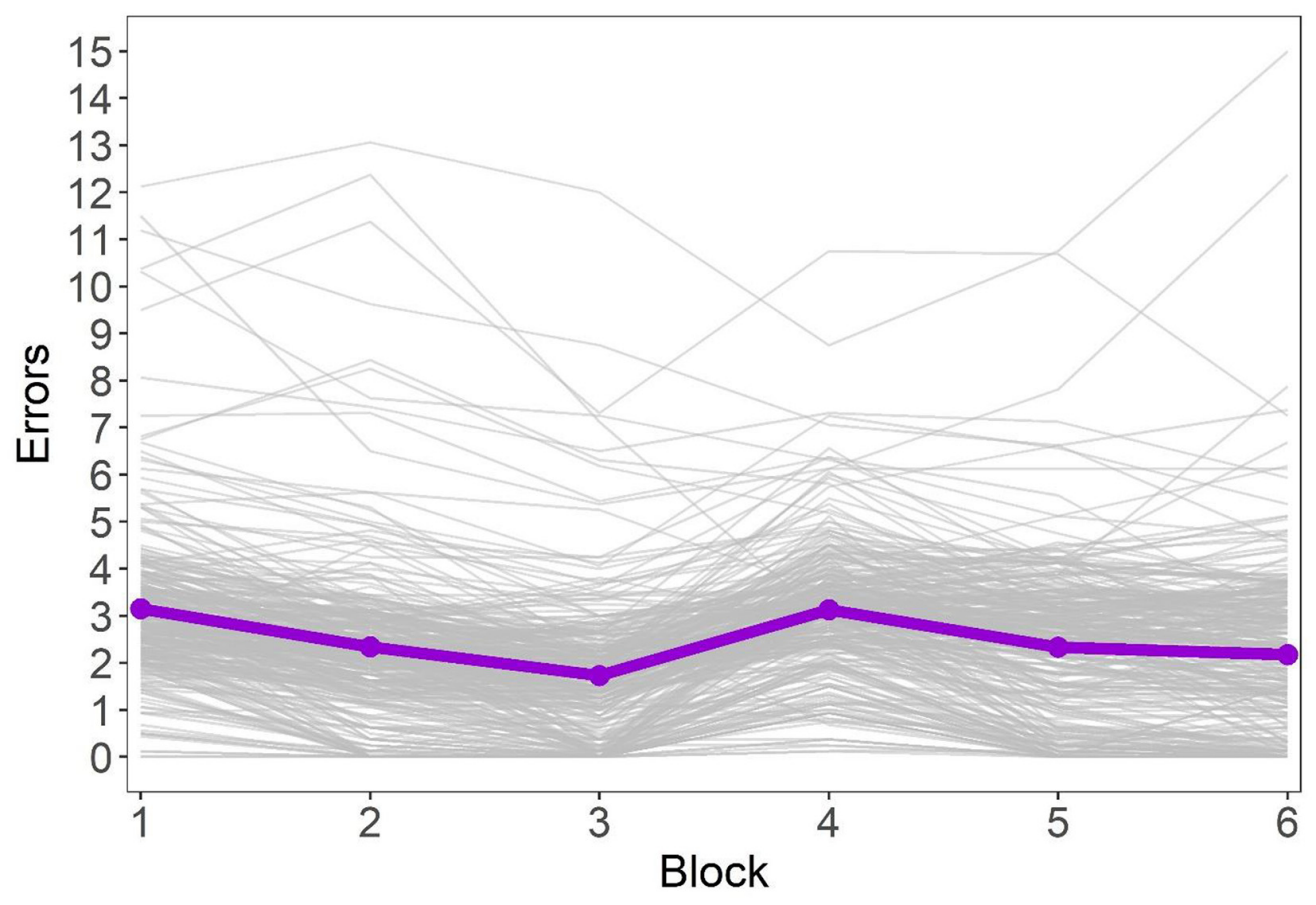
Participants' performance trajectories before the change (blocks 1–3) and after the change (blocks 4–6). The thin gray curves are individual trajectories (N = 300), and the wide violet curve represents the average performance on the task.

Importantly, individual performance trajectories can deviate from the average trajectory; people might have different scores at the beginning of the task, different learning rates, etc. The individual trajectories of the participants are displayed in Fig. 3, and visual inspection confirms substantial variability between people in the study. To account for the fact that some variability is due to real differences between people rather than measurement error, the variance of the latent change variables will also be estimated in the study. In total, three models will be considered.

The first model describes the average performance trajectory over the six measurement occasions. Next, two dummy variables containing information about the participant's assigned condition are introduced as predictors of TA, RA, and RA2 to examine the relevance of change communication. The control condition will be the reference group for both dummy variables. This approach ensures that each experimental group (one or two change‐related hints) is compared to the control condition. These direct comparisons enable one to examine to what extent change communication promotes adaptive performance.

The last research question will be examined by including task‐related state motivation as a predictor of performance on specific measurement occasions. Specifically, the first motivation (T1) assessment will be linked to the first performance score, the second motivation assessment (T2) will be connected to the performance scores on task blocks 2–4, and the third motivation assessment (T5) will be linked to the performance scores on the task blocks 5–6. This approach ensures that motivation assessment always precedes performance measurement, and each performance assessment is predicted by only one motivation predictor. The mean of the three motivation items was calculated for each measurement occasion. The scores were centered at the scale midpoint (4) and included as time‐varying predictors in the latent growth model. Since motivation was centered at the scale midpoint, the regression coefficients of motivation describe the difference in performance between people with state motivation at the scale midpoint and one point higher.

#### Transparency and openness

The study and the analyses were not pre‐registered. Data, codebook, all prior specifications, a list of packages, R code, and output are available at https://osf.io/x9dj5/.

## RESULTS

### Sample

Three hundred people were included in the final data set. Although 600 participants answered at least one question, some participants had to be excluded. Eighty participants abandoned the survey before completing the first attention check. One hundred twenty people were automatically excluded because they did not follow the attention check instructions. Nine participants did not complete the adaptive performance task and had to be excluded. Twenty failed the second attention check. Finally, 71 people did not show skill acquisition in the pre‐change phase of the task and were also excluded. Their performance at the end of the pre‐change phase (third block) was worse than at the beginning of the task (first block). However, participants with an excellent performance level (i.e., average error in the third block <1) were not excluded because they clearly understood the task, and small performance fluctuations can be viewed as artifacts. No participants had to be excluded because of other criteria, such as response patterns or speed.

### Descriptive statistics

**Fig. 4:**
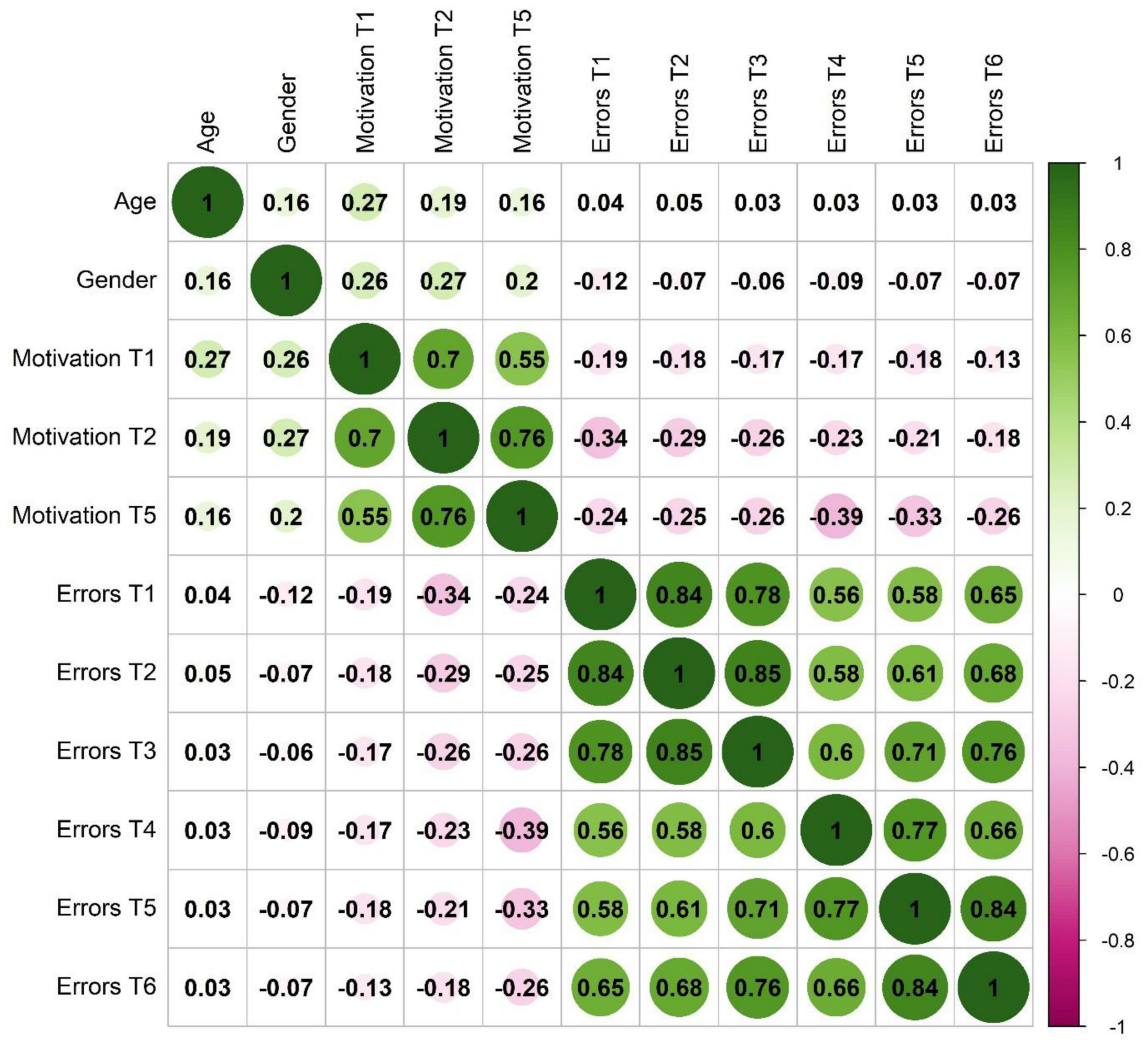
Bivariate correlations between demographic variables, task‐related state motivation, and task performance (T1‐T3 = pre‐change, T4‐T6 = post‐change). *N* = 300 except for gender (*N* = 299, 0 = female, 1 = male, without one person identifying as inter/diverse).

Descriptive statistics for the main variables and demographic data are provided in Tables 2 and 3. The correlation patterns are displayed in Fig. 4. The three change communication conditions were of similar size. The control condition (i.e., no change communication) consisted of 101 participants, the first experimental condition (i.e., change communication at the beginning of the task) consisted of 104 participants, and there were 95 people in the second experimental condition (i.e., change communication at the beginning of the task and directly after the change). Most participants identified as female (170 women, 129 men, and one intersex person). The participants' age varied between 18 and 74 (*M* = 32.68, *SD* = 12.20). 122 people (41%) had a college degree, and most participants had a job when completing the survey (227 people or 76%).

**Table 2:** Descriptive statistics (metric variables) for the whole sample and the three change communication conditions (CG/EG1/EG2)

	*M*	*SD*	Min	Max
Age	32.68 (32.40/32.64/33.03)	12.2 (11.77/12.23/12.73)	18 (18/18/18)	74 (65/74/65)
Motivation T1	5.33 (5.38/5.14/5.49)	1.08 (0.95/1.23/1.01)	1.33 (3/1.33/3)	7 (7/7/7)
Motivation T2	5.00 (4.97/4.91/5.14)	1.37 (1.35/1.48/1.28)	1 (1.33/1/1.67)	7 (7/7/7)
Motivation T5	4.76 (4.65/4.62/5.02)	1.49 (1.50/1.55/1.39)	1 (1/1/1)	7 (7/7/7)
Errors T1	3.15 (3.07/3.12/3.27)	1.65 (1.56/1.65/1.75)	0 (0.5/0/0)	12.12 (10.38/11.5/12.12)
Errors T2	2.34 (2.25/2.31/2.48)	1.77 (1.79/1.49/2.01)	0 (0/0/0)	13.06 (12.38/9.62/13.06)
Errors T3	1.73 (1.74/1.71/1.75)	1.51 (1.47/1.33/1.73)	0 (0/0/0)	12.00 (7.25/8.75/12.00)
Errors T4	3.13 (3.34/3.18/2.85)	1.41 (1.22/1.16/1.78)	0.12 (0.69/0.94/0.12)	10.75 (7.31/7.25/10.75)
Errors T5	2.33 (2.52/2.41/2.05)	1.67 (1.46/1.62/1.90)	0 (0/0/0)	10.75 (7.12/7.81/10.75)
Errors T6	2.17 (2.20/2.27/2.04)	1.85 (1.68/1.81/2.06)	0 (0/0/0)	15.00 (7.88/12.38/15.00)

*Note*: *N* = 300 (CG = 101, EG1 = 104, EG2 = 95); CG = control group (i.e., no change communication); EG1 = experimental condition that received a change‐related hint only at the beginning of the task; EG2 = experimental condition that received both an initial change‐related hint and a hint directly after the change; Motivation = task‐related state motivation (1 = does not apply, 7 = applies); Errors = mean task performance (0 = no errors, approx. 22 = worst answers on all trials) before the change (blocks 1–3) and after the change (blocks 4–6).

**Table 3:** Descriptive statistics (categorical variables) for the whole sample and the three change communication conditions (CG/EG1/EG2)

	Overall	CG	EG1	EG2
Gender				
Female	170 (57)	59 (58)	60 (58)	51 (54)
Inter	1 (0)	0 (0)	1 (1)	0 (0)
Male	129 (43)	42 (42)	43 (41)	44 (46)
Change noticed				
No	48 (16)	16 (16)	19 (18)	13 (14)
Yes	252 (84)	85 (84)	85 (82)	82 (86)
Task focus				
Accuracy	54 (18)	18 (18)	22 (21)	14 (15)
Speed	26 (9)	6 (6)	11 (11)	9 (9)
Both	205 (68)	74 (73)	68 (65)	63 (66)
Other	15 (5)	3 (3)	3 (3)	9 (9)
Task strategy				
Detected weights	70 (23)	24 (24)	20 (19)	26 (27)
Trying out numbers	16 (5)	6 (6)	6 (6)	4 (4)
Understand and plan	129 (43)	41 (41)	47 (45)	41 (43)
Using feedback	64 (21)	23 (23)	24 (23)	17 (18)
Other	21 (7)	7 (7)	7 (7)	7 (7)
Aid				
No	241 (80)	80 (79)	84 (81)	77 (81)
Yes	59 (20)	21 (21)	20 (19)	18 (19)

*Note*: Percentages are reported in brackets. *N* = 300 (CG = 101, EG1 = 104, EG2 = 95); CG = control group (i.e., no change communication); EG1 = experimental condition that received a change‐related hint only at the beginning of the task; EG2 = experimental condition that received both an initial change‐related hint and a hint directly after the change; All variables, except for gender, were assessed after completing the task (self‐report).

After completing the adaptive performance task, the majority of the sample reported that they noticed the task requirements change during the task (252 participants or 84%). Furthermore, 59 participants (20%) admitted using some aid, such as pen and paper, while completing the task. In both cases, there were no substantial differences between the three change communication conditions.

When asked about the speed‐accuracy‐tradeoff, most participants claimed they focused on both speed and accuracy (205 people or 68%). Fifty four participants (18%) focused primarily on the accuracy of their responses, and 26 people (9%) focused on speed. The remaining 15 participants claimed to use other strategies (e.g., changing their focus during the task). Interestingly, some conditions were more likely to use certain tradeoffs. Specifically, participants in the control condition were more likely to focus on both speed and accuracy (CG = 73%, EG1 = 65%, EG2 = 66%). In contrast, the proportion of people focusing solely on speed was the smallest in the control condition (CG = 6%, EG1 = 11%, EG2 = 9%). The second experimental condition had a higher proportion of people claiming to have used other strategies (CG = 3%, EG1 = 3%, EG2 = 9%).

There were also some differences concerning the strategy chosen to deal with the changed requirements. In particular, the second experimental condition was less likely to rely on feedback (CG = 23%, EG1 = 23%, EG2 = 18%), and they claimed more often that they focused on detecting the task weights (CG = 24%, EG1 = 19%, EG2 = 27%).

### Performance trajectories

Inspection of the individual trajectories (Fig. 3) and the results of the latent growth model containing the change variables (see Table 4) confirmed that the chosen task measured adaptive performance. Specifically, participants had, on average, an error score of $b_{\text{Intercept}}=3.18$ (CR 95% [3.02 to 3.34]) at the first measurement occasion, and the errors decreased during the pre‐change phase ($b_{\mathrm{SA}}=-0.97$, CR 95% [−1.09 to −0.86]). The greatest improvements were made early during the task ($b_{\mathrm{SA}2}=0.13$, CR 95% [0.07 to 0.18]).

**Table 4:** Results of the Bayesian latent growth models predicting performance (errors) on the task by change communication condition

Variable	Model 1 (basic)			Model 2 (change communication)		
	*b*	Error	CR 95%	*b*	Error	CR 95%
Means (latent variables)						
Intercept	3.18	0.08	[3.02 to 3.34]	3.19	0.09	[3.02 to 3.36]
Skill acquisition (SA)	−0.97	0.06	[−1.09 to −0.86]	−0.97	0.06	[−1.08 to −0.86]
Quadratic SA (SA2)	0.13	0.03	[0.07 to 0.18]	0.13	0.03	[0.07 to 0.18]
Transition adaption (TA)	1.40	0.06	[1.28 to 1.53]	1.56	0.09	[1.39 to 1.73]
Reacquisition adaption (RA)	−1.05	0.06	[−1.18 to −0.93]	−1.05	0.07	[−1.19 to −0.91]
Quadratic RA (RA2)	0.29	0.03	[0.23 to 0.35]	0.26	0.04	[0.19 to 0.33]
Regressions (criterion ~ predictor)						
TA ~ EG1				−0.11	0.13	[−0.37 to 0.15]
TA ~ EG2				−0.50	0.14	[−0.76 to −0.23]
RA ~ EG1				0.01	0.13	[−0.25 to 0.27]
RA ~ EG2				−0.03	0.13	[−0.29 to 0.23]
RA2 ~ EG1				0.04	0.06	[−0.07 to 0.16]
RA2 ~ EG2				0.08	0.06	[−0.03 to 0.20]
Variances (manifest variables)						
Errors T1	0.17	0.12	[0.00 to 0.41]	0.14	0.11	[0.00 to 0.37]
Errors T2	0.50	0.06	[0.38 to 0.62]	0.50	0.06	[0.39 to 0.61]
Errors T3	0.03	0.04	[0.00 to 0.13]	0.03	0.04	[0.00 to 0.14]
Errors T4	0.18	0.11	[0.00 to 0.41]	0.29	0.13	[0.03 to 0.55]
Errors T5	0.46	0.06	[0.34 to 0.58]	0.42	0.07	[0.29 to 0.55]
Errors T6	0.06	0.07	[0.00 to 0.24]	0.08	0.08	[0.00 to 0.29]
Variances (latent variables)						
Intercept	2.61	0.24	[2.17 to 3.11]	2.65	0.24	[2.21 to 3.14]
Skill acquisition (SA)	0.17	0.09	[0.01 to 0.34]	0.22	0.11	[0.02 to 0.46]
Quadratic SA (SA2)	0.02	0.02	[0.00 to 0.05]	0.02	0.02	[0.00 to 0.06]
Transition adaption (TA)	1.31	0.17	[1.00 to 1.65]	1.17	0.17	[0.84 to 1.52]
Reacquisition adaption (RA)	0.40	0.11	[0.20 to 0.66]	0.33	0.11	[0.13 to 0.56]
Quadratic RA (RA2)	0.02	0.02	[0.00 to 0.07]	0.02	0.02	[0.00 to 0.08]

*Note*: *N* = 300; CR = credibility interval limits (95%); full output (e.g., correlations between latent variables, chain diagnostics) is available at https://osf.io/x9dj5/; EG1 = dummy variable for the first experimental group (CG as the reference group); EG2 = dummy variable for the second experimental group (CG as the reference group).

Following the task requirements' change after the third task block, the average error score increased ($b_{\mathrm{TA}}=1.40$, CR 95% [1.28 to 1.53]). However, most participants were able to learn the modified task ($b_{\mathrm{RA}}=-1.05$, CR 95% [−1.18 to −0.93]). Similar to the pre‐change phase, the most substantial improvements were observed early in the post‐change phase ($b_{\mathrm{RA}2}=0.29$, CR 95% [0.23 to 0.35]).

### Change communication and adaptive performance

**Fig. 5:**
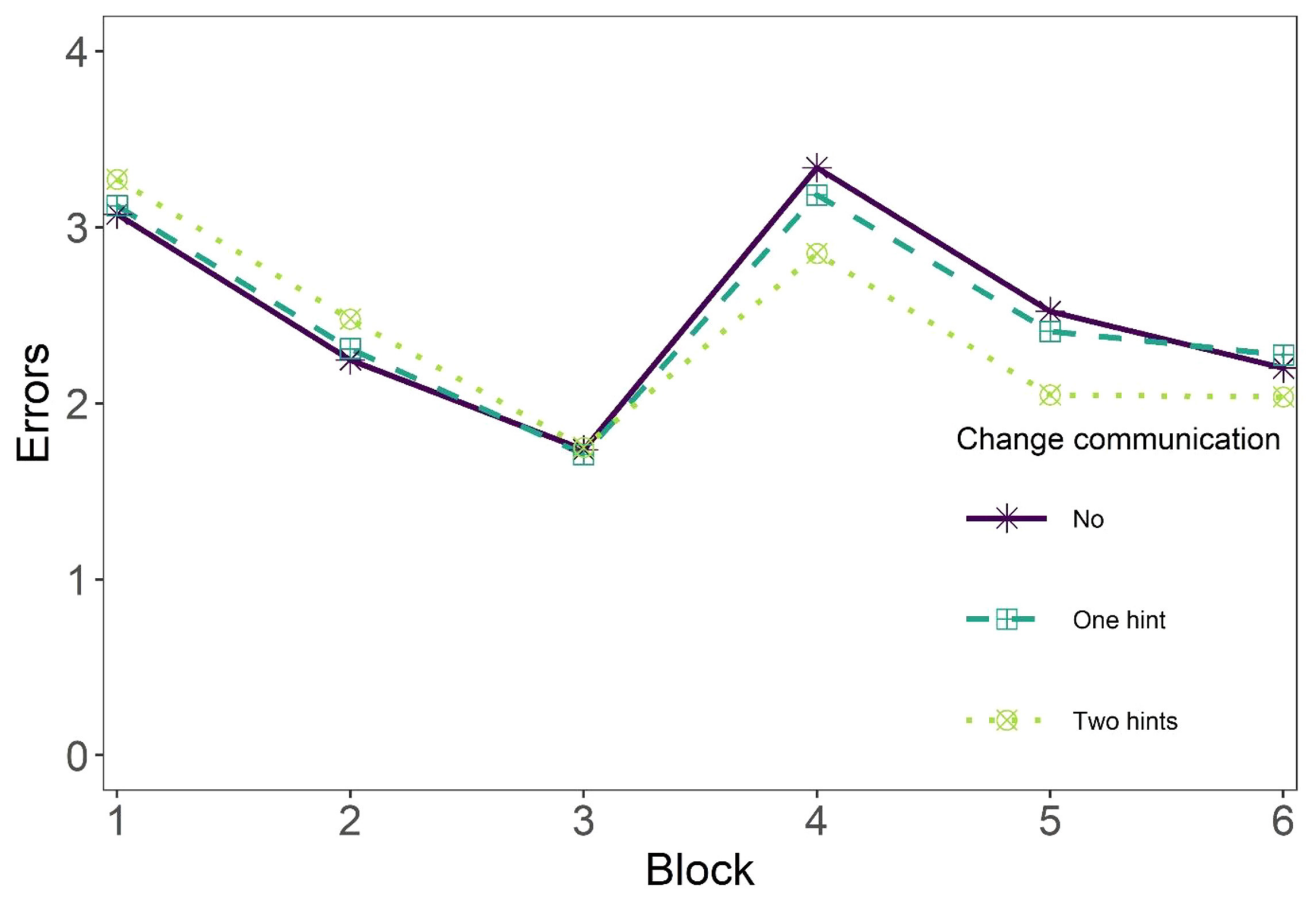
Differences between the change communication conditions (no communication vs. one hint at the beginning of the task vs. a hint at the beginning of the task and directly after the change). Presented are the average performance trajectories (*N* = 300) in the three conditions before the change (blocks 1–3) and after the change (blocks 4–6).

**Fig. 6:**
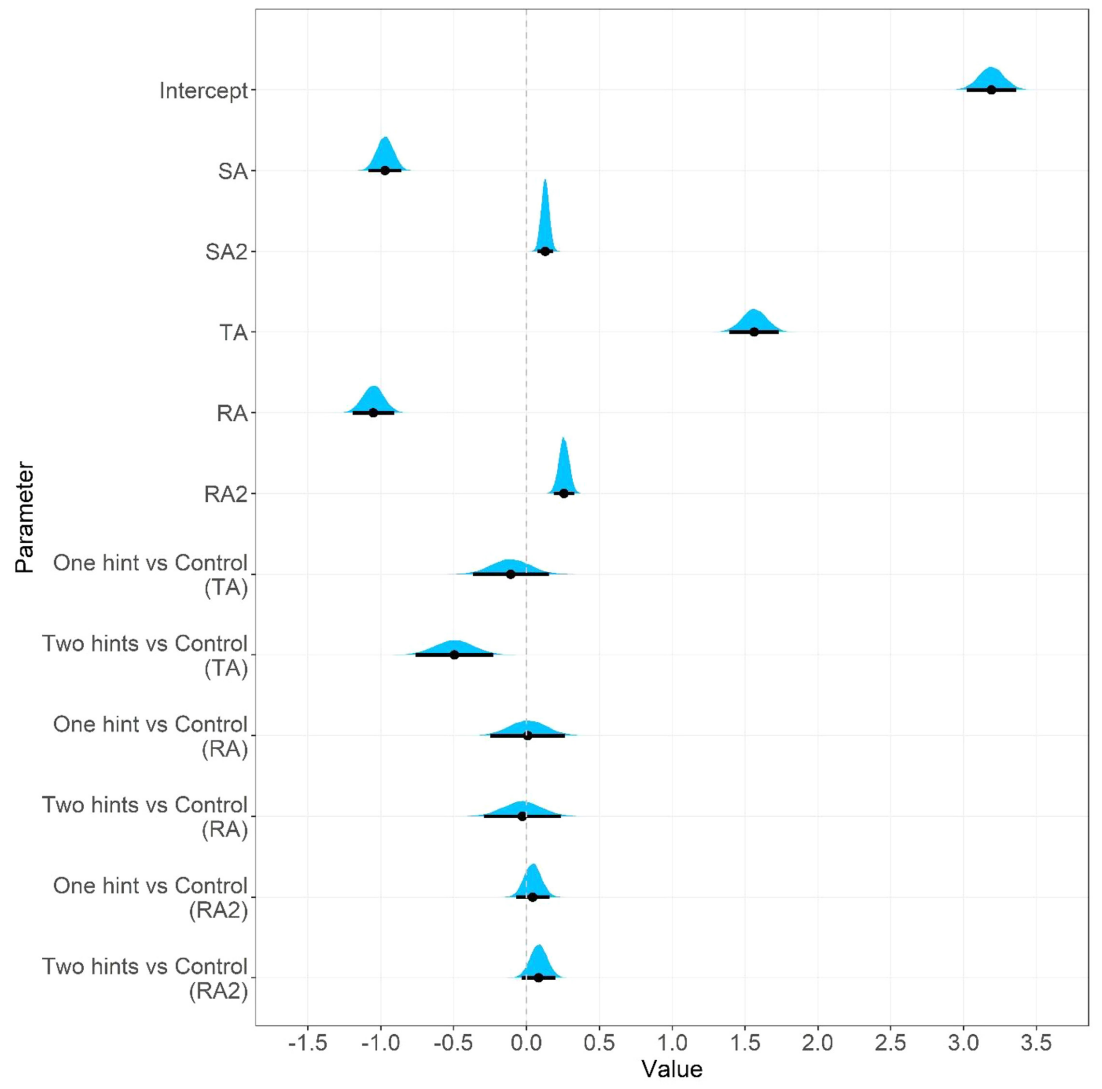
Posterior distributions for the relationship between group membership (change communication) and adaptive performance (*N* = 300). Black horizontal lines are 95% credibility intervals around the point estimates. Negative values in the group comparisons generally indicate that change communication is linked to better performance (i.e., fewer errors than in the control group). In the study, the group with two hints (i.e., one at the beginning of the task and one directly after the change) showed better adaptive performance immediately after the change (i.e., transition adaption).

To examine the role of change communication, group membership was added to the first model as a predictor of adaptive performance latent variable (TA, RA, RA2). The average performance trajectories of the three conditions (no change communication, single hint, two hints) are displayed in Fig. 5. A model summary is provided in Table 4, and the posterior distributions of the crucial coefficients are shown in Fig. 6.

As expected, the experimental group that received two change‐related hints (i.e., change communication at the beginning of the task and directly after the change) made smaller errors immediately after the change than the control group ($b_{\mathrm{TA}\_\mathrm{EG}2\_\mathrm{CG}}=-0.50$, CR 95% [−0.76 to −0.23]). The experimental group was largely able to maintain the advantage in the later stages because the learning rate was similar across both groups ($b_{\mathrm{RA}\_\mathrm{EG}2\_\mathrm{CG}}=-0.03$, CR 95% [−0.29 to 0.23]). However, on the last task block, the performance in the second experimental group plateaued (see Fig. 5), whereas the control group continued to improve their performance, which is reflected by the fact that the credibility interval for the quadratic comparison included mostly positive values ($b_{\mathrm{RA}2\_\mathrm{EG}2\_\mathrm{CG}}=0.08$, CR 95% [−0.03 to 0.20]).

There was no substantial evidence that a single change‐related hint provided at the beginning of the task facilitated transition adaption ($b_{\mathrm{TA}\_\mathrm{EG}1\_\mathrm{CG}}=-0.11$, CR 95% [−0.37 to 0.15]) or reacquisition adaption ($b_{\mathrm{RA}\_\mathrm{EG}1\_\mathrm{CG}}=0.01$, CR 95% [−0.25 to 0.27], $b_{\mathrm{RA}2\_\mathrm{EG}1\_\mathrm{CG}}=0.04$, CR 95% [−0.07 to 0.16]). Although most of the values in the TA posterior distribution were negative, they were mostly small. Visual inspection of Fig. 5 confirms that there were only minuscule differences between the average trajectories of these two conditions.

### State motivation and performance

**Fig. 7:**
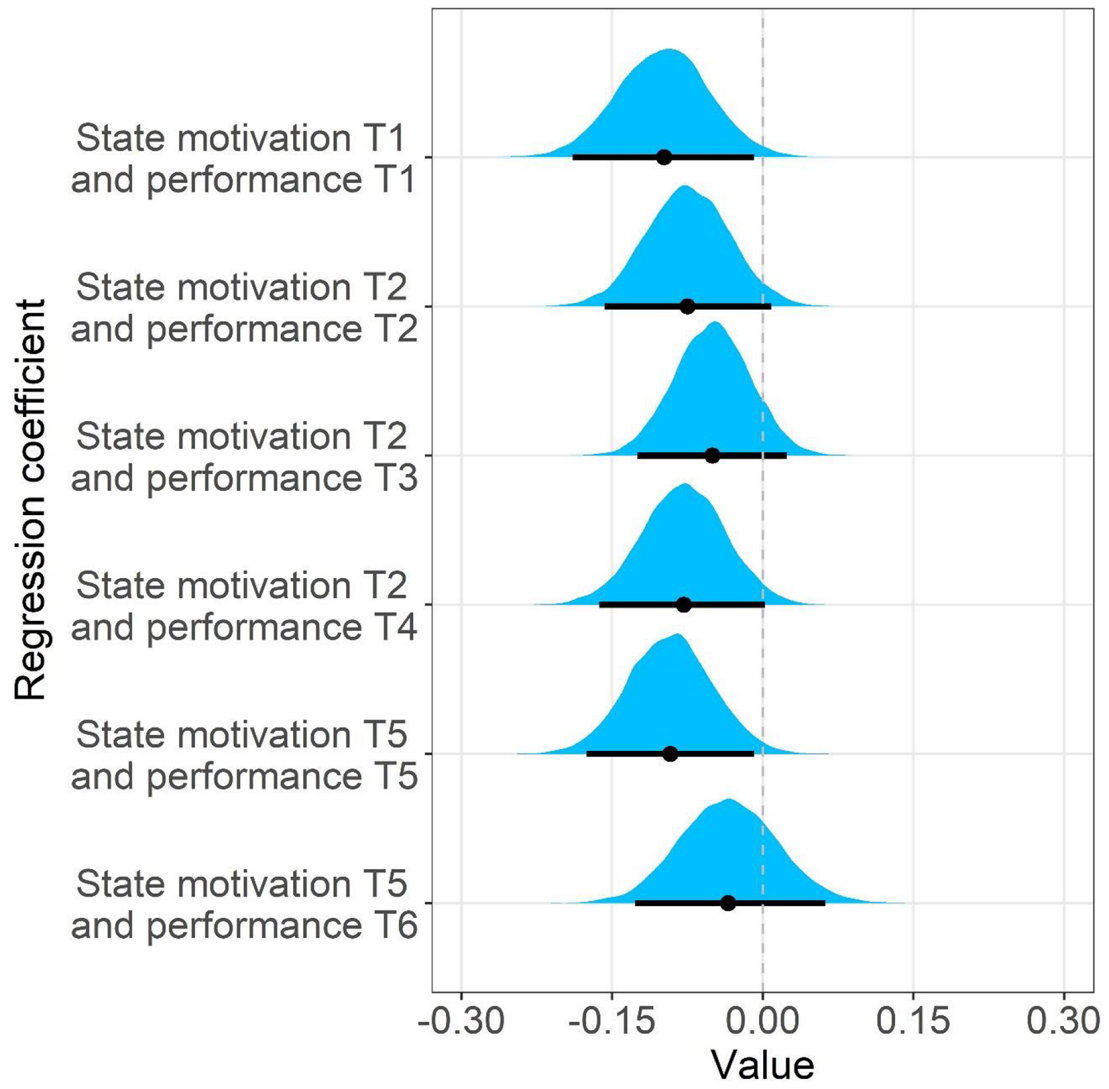
Posterior distributions for the relationship between task‐related state motivation and task performance (*N* = 300). Black horizontal lines are 95% credibility intervals around the point estimates. Negative values indicate that higher motivation is linked to better performance (i.e., fewer errors).

The three available state motivation measurement occasions were added to the baseline model to examine task‐related state motivation as a proximal predictor of performance. Since there were only three motivation assessments and six task blocks, some motivation scores were used to predict more than one performance score. Table 5 includes the model summary, and Fig. 7 contains the posterior distributions of the most relevant coefficients.

**Table 5:** Results of the Bayesian latent growth models predicting performance (errors) on the task by state motivation

Variable	Model 1 (basic)			Model 3 (state motivation)		
	*b*	Error	CR 95%	*b*	Error	CR 95%
Means (latent variables)						
Intercept	3.18	0.08	[3.02 to 3.34]	3.29	0.10	[3.10 to 3.48]
Skill acquisition (SA)	−0.97	0.06	[−1.09 to −0.86]	−1.01	0.07	[−1.14 to −0.88]
Quadratic SA (SA2)	0.13	0.03	[0.07 to 0.18]	0.13	0.03	[0.07 to 0.19]
Transition adaption (TA)	1.40	0.06	[1.28 to 1.53]	1.43	0.07	[1.29 to 1.57]
Reacquisition adaption (RA)	−1.05	0.06	[−1.18 to −0.93]	−1.05	0.07	[−1.19 to −0.92]
Quadratic RA (RA2)	0.29	0.03	[0.23 to 0.35]	0.28	0.03	[0.21 to 0.34]
Regressions (criterion ~ predictor)						
Errors T1 ~ Motivation T1				−0.10	0.05	[−0.19 to −0.01]
Errors T2 ~ Motivation T2				−0.07	0.04	[−0.16 to 0.01]
Errors T3 ~ Motivation T2				−0.05	0.04	[−0.13 to 0.02]
Errors T4 ~ Motivation T2				−0.08	0.04	[−0.16 to 0.00]
Errors T5 ~ Motivation T5				−0.09	0.04	[−0.18 to −0.01]
Errors T6 ~ Motivation T5				−0.03	0.05	[−0.13 to 0.06]
Variances (manifest variables)						
Errors T1	0.17	0.12	[0.00 to 0.41]	0.16	0.12	[0.00 to 0.41]
Errors T2	0.50	0.06	[0.38 to 0.62]	0.50	0.06	[0.38 to 0.61]
Errors T3	0.03	0.04	[0.00 to 0.13]	0.03	0.04	[0.00 to 0.14]
Errors T4	0.18	0.11	[0.00 to 0.41]	0.32	0.14	[0.05 to 0.58]
Errors T5	0.46	0.06	[0.34 to 0.58]	0.42	0.07	[0.28 to 0.55]
Errors T6	0.06	0.07	[0.00 to 0.24]	0.08	0.09	[0.00 to 0.30]
Variances (latent variables)						
Intercept	2.61	0.24	[2.17 to 3.11]	2.56	0.24	[2.12 to 3.05]
Skill acquisition (SA)	0.17	0.09	[0.01 to 0.34]	0.21	0.11	[0.01 to 0.44]
Quadratic SA (SA2)	0.02	0.02	[0.00 to 0.05]	0.02	0.02	[0.00 to 0.06]
Transition adaption (TA)	1.31	0.17	[1.00 to 1.65]	1.18	0.18	[0.86 to 1.55]
Reacquisition adaption (RA)	0.40	0.11	[0.20 to 0.66]	0.32	0.11	[0.12 to 0.55]
Quadratic RA (RA2)	0.02	0.02	[0.00 to 0.07]	0.02	0.02	[0.00 to 0.08]

*Note*: *N* = 300; motivation = task‐related state motivation, which was centered at scale midpoint (4); CR = credibility interval limits (95%); full output (e.g., correlations between latent variable, chain diagnostics) is available at https://osf.io/x9dj5/.

As expected, state motivation tended to be related to the performance on the next task block. On average, higher motivation was linked to better performance (i.e., fewer errors) on the first task block ($b_{\mathrm{MT}1\_\mathrm{PT}1}=-0.10$, CR 95% [−0.19 to −0.01]), the second task block ($b_{\mathrm{MT}2\_\mathrm{PT}2}=-0.07$, CR 95% [−0.16 to 0.01]), and the fifth task block ($b_{\mathrm{MT}5\_\mathrm{PT}5}=-0.09$, CR 95% [−0.18 to −0.01]). State motivation also tended to be related to performance on later task blocks, but the relationships were generally substantially smaller (see Fig. 7).

## DISCUSSION

Because the modern workplace is dynamic, organizations require guidance about change management processes and employee training. Since previous studies focused mainly on trait‐like predictors of adaptive performance, the main goal of the study was to examine more proximal antecedents of the adaption process. Specifically, the role of change communication and task‐related state motivation was considered. Overall, informing participants that the task requirements had changed resulted in smaller performance impairment in the first task block directly after the change. Furthermore, task‐related state motivation was related to performance on the next task block. The respective findings and their implications are discussed in the following sections.

### Change communication

In the study, some participants received an early hint at the beginning of the task about possible task changes due to the market crash and an additional hint directly after the change. Providing the two hints resulted in smaller performance impairment or better transition adaption than in the control condition, which did not receive change‐related information. In contrast, providing a hint only at the beginning of the task did not lead to substantially better transition adaption. Furthermore, in both experimental conditions, hints did not substantially improve the learning rate in the later stages of the task (i.e., reacquisition adaption).

The finding that very early change communication is insufficient to improve adaption substantially can be seen as an extension of previous findings (Bröder & Schiffer, 2006). While Bröder and Schiffer showed that a moderately informative hint provided after the change is not necessarily of substantial help, the findings indicate that a more informative hint (i.e., communicating which task aspects will change) provided much earlier might also have negligible effects. Thus, not only the informativeness of the hint but also the timing of change communication must be considered. This conclusion is corroborated by the findings related to the second experimental condition. Specifically, an additional informative hint provided directly after the change proved helpful, as people in this condition made fewer errors during the transition phase than in the control condition. This finding is important because previous studies demonstrating the usefulness of change communication used subjective performance ratings rather than objective performance data (Parent *et al*., 2012; Petrou *et al*., 2018; Van den Heuvel *et al*., 2013). Overall, the results indicate that organizations should consider informing employees about changes to mitigate change‐related performance impairment and associated financial costs. Moreover, the findings corroborate the process model of adaptive performance (Jundt & Shoss, 2023), as they demonstrate the significant role of change detection (e.g., through change communication) in initializing the adaption process. Notably, it appears that change communication needs to be informative (e.g., providing some details about what will change) and well‐timed (e.g., change communication shortly before or after the change).

Notwithstanding the importance of change communication to transition adaption, it is important to note that there was no substantial relationship with the learning rate in the later stages of the study (i.e., reacquisition adaption). In other words, the learning rate in the experimental conditions looked similar to that in the control condition. However, these findings should be viewed together with the results concerning transition adaption. Since a single early hint did not facilitate transition adaption, it is not surprising that it also did not improve adaption in the later stages. While the lack of a substantial relationship in the second experimental condition might seem surprising at first, the pattern of the results is plausible. Thanks to the additional hint, people in the second experimental group could minimize performance impairment directly after the change, which means less space for improvement in the later stages compared to the control condition. Nonetheless, since the learning rate was similar in both conditions, the second experimental condition largely maintained its advantage throughout the post‐change phase. Thus, even if change communication facilitates transition but not reacquisition adaption, it still offers a medium‐term advantage.

The average change‐induced performance drop in the control condition was approximately 1.6 points (see Table 2), and the performance level of the experimental condition with the additional hint was better by approximately 0.5 points. Thus, providing a moderately informative hint after the change helped improve performance by almost 30%. Such improvements could translate to substantial savings for organizations. Therefore, organizations should consider informing employees about changes in task‐related requirements.

Further research is needed to make more specific recommendations for practitioners about the required informativeness of change communication in dynamic settings and the optimal timing of providing change information. Since a moderately informative change‐related hint did not improve adaption substantively in a previous study (Bröder & Schiffer, 2006) and a more informative hint was linked to a performance improvement of approximately 30% in the study, one could examine whether more specific hints provide an additional advantage. To illustrate, one could inform employees what aspects of the task changed and to what extent. Such prior knowledge could help people discard unreasonable strategies more quickly. However, it is important to avoid information overload when providing change‐related hints; providing too much information might be harmful. To illustrate, there is some evidence that providing a summary of past decisions might impair the adaption process (Rakow & Miler, 2009).

Concerning the timing of change communication, it seems that providing an informative hint shortly before or after the change can be helpful for specific tasks. However, in the organizational context, the timing of changes cannot always be controlled, which raises the question of whether informing people about possible changes in the near future can be helpful. While organizations cannot predict all changes (e.g., changes due to the COVID‐19 pandemic), communicating planned changes in organizations might mitigate losses, such as materials, money, or even lives.

According to the study, providing a moderately informative hint well in advance might not be enough to facilitate transition adaption. Future studies could examine whether providing an even more specific hint in advance can be more beneficial. Furthermore, one could investigate the role of supportive communication during task completion because a supportive environment (e.g., managers) is regarded as one of the factors affecting people's reactions to changes (Oreg *et al*., 2011). In addition, one could investigate to what extent periodically repeating a hint might increase change readiness and facilitate the adaption process. According to Bröder & Schiffer (2006), some people may forget a single hint or not pay attention to the task instructions. Thus, repetition could increase the chance that change will be registered faster, which could facilitate transition adaption.

Future studies could also examine which groups benefit the most from change communication. Since cognitive abilities are related to objectively measured adaptive performance (Stasielowicz, 2020), one could investigate whether providing hints about simple changes to people with high cognitive abilities leads to any noticeable improvements. It is possible that communicating simple changes could cause irritation and lead to unnecessary interruptions. Thus, it is recommended to examine whether the benefits of change communication exceed the costs or whether a more nuanced approach that entails tailoring change communication to the individual characteristics of the employees is needed.

Furthermore, it is recommended to investigate whether well‐timed change communication improves team adaption to a similar extent as individual adaption. After all, many job tasks go beyond individual adaptive performance and require team coordination. As groups need to coordinate their actions, it is not surprising that the importance of within‐team communication in dynamic contexts has been acknowledged in the extant literature (Burke, Stagl, Salas, Pierce & Kendall, 2006; Maynard, Kennedy & Sommer, 2015). However, examining the role of providing change‐related information in advance was neglected in the past. Since groups' and individuals' performance trajectories sometimes follow different patterns (Lejarraga, Lejarraga & Gonzalez, 2014), it cannot be assumed that change communication will have similar effects on team adaptation.

Further work is also needed to delineate the mechanism linking change communication to better adaptive performance. According to the model proposed by Jundt and Shoss (2023), detecting or anticipating change, identifying change‐related demands, and developing strategies are the first steps of the adaption process. One could speculate that change communication helps recognize faster that old strategies must be modified to avoid extensive performance impairment. Realizing that the environment has changed could motivate people to increase their efforts to find better strategies. For example, people might scan the environment for change‐related cues or develop a systematic plan to deal with the change. Descriptive statistics in the study confirm that the individual approaches depend somewhat on the available change‐related information (Table 3). The group that received two change‐related hints was less likely to rely on task feedback than the control group (EG2 = 18% vs. CG = 23%). Instead, they were more likely to report that they detected the weights underlying the task (EG2 = 27% vs. CG = 24%). However, it is important to note that the strategies were assessed after completing the task. Furthermore, the differences are not large. To offer more conclusive evidence about strategy use, one could use process‐tracing methods (Schulte‐Mecklenbeck, Kühberger & Johnson, 2019), such as thinking aloud and EEG, to collect direct evidence about change perception and strategies deployed during the adaption process.

It is also possible that change communication influences adaptive performance through various emotional, cognitive, and motivational processes. Since experiencing change might be stressful and requires re‐allocation of attentional resources (Jundt & Shoss, 2023), it is worth examining to what extent change communication affects these processes. For example, change communication might influence metacognition, which helps people guide their behavior in changing and complex environments (Fischer, Huff, Anders & Said, 2023). For example, some studies have linked metacognitive activities, such as planning and goal monitoring to better adaptive performance (Christian *et al*., 2017; Jundt *et al*., 2015). Furthermore, providing change‐related information could increase motivation, which helps maintain relatively good performance levels. This possibility will be discussed in more detail after summarizing the findings pertaining to the study's second research question: the relationship between state motivation and performance.

### Task‐related motivation

As expected, state motivation was related to performance on the next task block but not necessarily later task blocks in this study. This pattern confirms that temporal proximity needs to be considered when examining predictors of adaptive performance. In future studies, one could assess motivation before each task block instead of before selected blocks. The task consisted of six stimuli blocks, and the decision was made to measure motivation only three times: before the task, after the first block, and directly after the change. This procedure ensured that participants could learn the task before the change occurred, as they did not experience frequent interruptions. However, in one study examining adaptive performance, time‐varying covariates were assessed after each task session (Jorgensen *et al*., 2021; Richels, Day, Jorgensen & Huck, 2020). A similar approach could be adopted in future studies. After all, in this study, none of the participants mentioned interruptions in their open comments, and the individual performance trajectories looked similar to those from other studies (Howe, 2019; Lang & Bliese, 2009). This indicates that learning the task was possible, and one could consider increasing the number of interruptions to collect more information about time‐varying covariates in future adaptive performance studies.

Including more measurements of time‐varying covariates would also facilitate building more complex models that enable disaggregating between‐person and within‐person effects of predictors. Since five or more waves of measurements are recommended to obtain precise estimates of predictor effects for simple linear trajectories (Hori & Miyazaki, 2022, 2023), it can be safely assumed that less than five measurements will probably not be enough when modeling complex trajectories like nonlinear adaption processes. At the same time, researchers need to consider the task duration and burden for the participants. Instead of cramming relatively long tasks and multiple measures of covariates into one session, one could consider spacing out the measurement occasions across one whole working day, several days, or even weeks.

The finding that higher task‐related state motivation corresponds to better performance has implications for organizations often dealing with dynamic situations. While the magnitude of the overall motivation effect on a particular task block is relatively small, this can quickly add up to greater overall effects across multiple measurement occasions. Therefore, practitioners might want to ensure that task‐related motivation does not decline substantially across the whole task. Since several articles reviewing motivational interventions are available, organizations could use this literature to guide their decisions. To illustrate, providing one‐on‐one consultations (Slemp, Lee & Mossman, 2021) or describing task value to the employees can help maintain motivation (Harackiewicz & Priniski, 2018).

Overall, current findings are consistent with the adaptation process model, which posits that multiple emotional, cognitive, and motivational variables can be linked to the different stages of the adaption process (Jundt & Shoss, 2023). In previous studies, metacognitive activities, such as planning or goal monitoring, were linked to better adaptive performance (Jundt *et al*., 2015), and higher affect variability corresponded to worse performance (Richels *et al*., 2020). Based on the findings, task‐related state motivation can be added to the growing list of proximal adaptive performance predictors.

The study also offers preliminary evidence that change communication helps maintain motivation and subsequent effort. Overall, task‐related state motivation tended to be above the scale midpoint (4), but it declined during the task ($M_{T1}=5.33$, $M_{T2}=5.00$, $M_{T5}=4.76$). However, the group that received two change‐related hints reported higher motivation than the control group after the change ($M_{T5\_\mathrm{EG}2}=5.02$ vs. $M_{T5\_\mathrm{CG}}=4.65$). A similar trend was observed when using an alternative motivation measure (0–100) consisting of one item ($M_{T5\_\mathrm{EG}2}=72.62$ vs. $M_{T5\_\mathrm{CG}}=65.49$). Thus, in future studies, one could systematically examine whether motivation mediates the effect of change communication on performance. While the Bayesian approach used is relatively flexible, such complex models could yield very uncertain estimates that manifest in very wide credibility intervals. To ensure that the respective estimates are useful, it is recommended to adjust the design (e.g., reduce the number of conditions or use larger samples) and priors (e.g., use more informative priors based on the available results) in future studies.

The findings confirm that including proximal predictors of adaptive performance can be fruitful. While replication studies examining predictors included in this study (i.e., change communication and motivation) and previous studies (e.g., affect) should be encouraged, researchers could also consider accounting for other cognitive, emotional, and motivational processes that can be connected with different stages of the adaption process (Jundt & Shoss, 2023). One such example is effort, which can be viewed as an even more proximal performance predictor than motivation. Work effort or engagement can translate into putting more energy into the task and increasing the intensity of one's action (Van Iddekinge *et al*., 2023), which could be helpful in dynamic contexts. In particular, a high effort could be beneficial when identifying change‐related demands or developing, implementing, and revising strategies.

## LIMITATIONS

While this study helps advance the research field by examining proximal predictors of adaptive performance, it is important to acknowledge the limitations of the current research. Notwithstanding the advantages of objective measures of adaptive performance, it is important to note that a single task cannot tap into all adaptive performance dimensions simultaneously. The current task captures several aspects proposed in the original taxonomy (Pulakos *et al*., 2000; Pulakos, Schmitt, Dorsey, Arad, Hedge & Borman, 2002), such as dealing with uncertain or unpredictable situations, learning new tasks or procedures, and, to a certain extent, handling emergencies or crises. However, the task does not assess dimensions like physical adaptive performance or intercultural adaptive performance. Considering that such aspects might be relevant for some jobs, such as soldiers, athletes, or astronauts, research using other tasks is needed to corroborate the positive effects of change communication and task‐related state motivation in those occupational areas.

More research is also required to improve our understanding of the mechanisms underlying the positive effects of change communication. The study cannot offer a conclusive description of the mechanism in the experimental condition, which received both a very early hint and an additional hint directly after the change. Specifically, it is unclear whether improved transition adaption can be attributed to the timing of the second hint, the “repetition” of the hint, or both. To answer this research question, a fourth condition would be required, including a hint after the change but no hint at the beginning of the task. However, with three conditions, the design was already relatively complex, and the decision was made to examine whether providing a hint could be helpful at all. This decision was motivated by the fact that a study using a less informative hint (Bröder & Schiffer, 2006) found no substantial benefits in a dynamic context. By choosing a relatively informative hint and repeating it directly after the change in the study, the chances of observing the benefits of change communication were increased. Indeed, only the condition that received both hints benefited substantially from change communication. As noted in the preceding sections, future research should examine the role of timing, the importance of repeating the hint periodically and increasing the informativeness of the hint even further.

Another point that needs to be addressed in future studies pertains to modeling the time‐varying covariates. Even though the study provides evidence that task‐related state motivation can be relevant in dynamic contexts, it cannot offer estimates of disambiguated within‐person and between‐person effects. To estimate such effects precisely, more than three measurements of state motivation would be required (Hori & Miyazaki, 2022, 2023). Furthermore, it would require including additional latent variables to model motivation effects. The models already contain six latent variables and 15 covariances between latent variables to model the adaptive performance trajectories. In total, approximately 40 parameters were estimated using data from 300 participants to answer the research question. Including more latent variables would imply even a smaller participants‐to‐parameters ratio and could yield unreliable results. While the Bayesian approach enables one to compensate for relatively small sample sizes by using informative priors, examining a more complex model would require very informative priors, which are hard to justify because they can strongly influence the results. Therefore, larger samples are recommended to accommodate even more latent variables and avoid using unjustifiably strong priors in such models. At the same time, the current results can be used to justify slightly more informative priors in future studies.

In addition, the plausibility of causal conclusions derived from the study must be addressed. Notably, the randomization of change communication and the repeated measures design of the task increases the plausibility of causal explanations. In particular, the total effect of change communication on the adaption process is expected to be accurate to a large extent. However, further research is needed to estimate direct and indirect effects accurately. To illustrate, since the study contains preliminary evidence that change communication helps maintain motivation, one could argue that it is necessary to account for motivation when estimating the direct effect of change communication. Other mediating variables are also possible, including affect (Jorgensen *et al*., 2021; Jundt & Shoss, 2023; Richels *et al*., 2020) and metacognition (Jundt *et al*., 2015).

As to the causal effects of motivation, randomization was not possible for motivation, which means that additional variables could have impacted the effect estimates of motivation. One potentially relevant variable is change communication, which seems to be related to both motivation and performance. Therefore, testing more complex models with very large samples was suggested in the preceding paragraphs to provide accurate effect estimates. However, it cannot be ruled out that there are unknown confounding variables that might be worth adjusting for (Cinelli, Forney & Pearl, 2022). To illustrate, since the study was conducted online during the COVID‐19 pandemic, some uncontrolled environmental factors, such as drilling, lawnmowers, or other noises at home, could affect motivation and performance. Therefore, one could conduct similar studies in laboratories to reduce the number of potential confounding variables and help estimate accurate causal effects of task‐related state motivation.

Finally, while the reliability of the state motivation measure was acceptable on all three measurement occasions (≥0.71), future studies might consider using other motivation measures. One possibility to increase reliability is to use longer measures. In the study, a three‐item was chosen to avoid distracting participants from the main task and reduce the general participant burden. However, as indicated in earlier sections, there is no evidence that interruptions negatively affected task performance. Thus, one could consider using slightly longer motivation measures in future studies. Another option would be to use measures focusing on a single motivation aspect rather than trying to assess various motivational elements, such as self‐efficacy and experiencing positive valence when completing a task.

## CONCLUSION

Previous adaptive performance studies seeking to offer guidance about change management and employee training focused primarily on identifying trait‐like predictors of adaptive performance, such as cognitive ability and conscientiousness. This study advances the research field by examining two proximal predictors – change communication and task‐related state motivation. Providing informative change‐related hints at the beginning of a dynamic task and shortly after the change led to better transition adaption. Specifically, it resulted in smaller performance impairment directly after the change. Crucially, the performance advantage was maintained to a large extent in the relearning phase of the task. In addition, task‐related state motivation was related to the performance on the next task block. The findings indicate that organizations could benefit from using informative and timely change communication to improve employee performance. Future studies can investigate the specific mechanism of change communication's positive effects; the mediating variables might include state motivation and metacognition.

I am grateful to Lisa Herbig for the help with survey implementation and data collection. The acknowledgment does not imply the endorsement of the views presented in the current manuscript. The author remains solely responsible for the views expressed in this study. This research received no specific grant from any funding agency in the public, commercial, or not‐for‐profit sectors. The authors declare that they have no conflict of interest. The project was carried out in accordance with the ethical guidelines of the American Psychological Association. The participants provided informed consent based on the official template of the local ethics committee (Osnabrück University). The study materials, data, and analysis scripts used for this article can be accessed at https://osf.io/x9dj5/.
